# Supplementary material for: The role of metabolism in shaping enzyme structures over 400 million years
Source: Nature. 2025 Jul 9;644(8075):280–9. doi: 10.1038/s41586-025-09205-6 (PMC12328220; doi:10.1038/s41586-025-09205-6)
Supplement: Supplementary file 1 — Legends for Supplementary Tables 1–10, Notes, Methods and additional references. [file 41586_2025_9205_MOESM1_ESM.pdf]

nature portfolio

https://doi.org/10.1038/s41586-025-09205-6

Supplementary information

The role of metabolism in shaping enzyme structures over 400 million years

In the format provided by the authors and unedited

Supplementary Information For:

The role of metabolism in shaping enzyme structures over 400 million years

Oliver Lemke[1,2,A], Benjamin Murray Heineike[1,3,4,A], Sandra Viknander[5], Nir Cohen[1], Feiran Li[5], Jacob Lucas Steenwyk[6,7,8,9], Leonard Spranger[1], Federica Agostini[1], Cory Thomas Lee[1], Simran Kaur Aulakh[3,4], Judith Berman[10], Antonis Rokas[7,9], Jens Nielsen[5], Toni Ingolf Gossmann[11], Aleksej Zelezniak[5,12,13], Markus Ralser[1,2,3,4,14,*]

[1] Department of Biochemistry, Charité - Universitätsmedizin Berlin, Berlin, Germany
[2] Exploratory Diagnostic Sciences, Berlin Institute of Health at Charité, Berlin, Germany
[3] Center for Human Genetics, Nuffield Department of Medicine, University of Oxford, Oxford, UK
[4] Molecular Biology of Metabolism Laboratory, The Francis Crick Institute, London, UK
[5] Department of Life Sciences, Chalmers University of Technology, Gothenburg, Sweden
[6] Howard Hughes Medical, University of California, Berkeley, Berkeley, CA, USA
[7] Department of Biological Sciences, Vanderbilt University, Nashville, TN, USA
[8] Department of Molecular and Cell Biology, University of California, Berkeley, Berkeley, CA, USA
[9] Evolutionary Studies Initiative, Vanderbilt University, Nashville, TN, USA
[10] Shmunis School of Biomedical and Cancer Research, George S. Wise Faculty of Life Sciences, Tel Aviv University,Ramat Aviv, Israel
[11] Faculty of Biochemical and Chemical Engineering, TU Dortmund University, Dortmund, Germany
[12] Institute of Biotechnology, Life Sciences Centre, Vilnius University, Vilnius, Lithuania
[13] Randall Centre for Cell & Molecular Biophysics, King's College London, London, UK
[14] Max Planck Institute for Molecular Genetics, Berlin, Germany

[A] Equally contributed to the work
*
Corresponding author. Email: markus.ralser@charite.de

Table of Contents

Table of Contents **2**
Supplemental Tables **3**
Supplementary Notes **4**
Note 1: Choice of Saccharomycotina yeasts for addressing metabolic evolution 4
Note 2: Limitations of AlphaFold2 in relation to our study 4
Note 3: Further details on specific hypotheses generated from structural analysis. 6
Note 3.1: Xylose pathway specialization 6
Note 3.2: Hsu1p/Str2p structural divergence 7
Note 4: Description of cost metrics 7
Note 5: Observations that require known structures 8
Note 6: Investigating signals of evolutionary selection in enzyme evolution 10
Note 6.1: Higher degree of evolutionary selection in some orthogroups 10
Note 6.2: Evolutionary selection affects structural elements differently 11
Methods **13**
Orthogroup and Species Selection 13
Structural predictions using AlphaFold2 14
Multiple structure alignment and Orthogroup refinement 15
Multiple sequence alignments and phylogenetic trees 17
Metabolic network visualisation 18
Phenotype selection 18
Determining protein abundance using mass spectrometry 19
Cultivation 19
Cell lysis 19
Sample preparation for mass spectrometry 20
Liquid chromatography mass spectrometry 21
Data processing 21
Mapping structures within an orthogroup 22
Binding site extraction 22
Protein-protein interaction site extraction 23
Structural information extraction 23
Estimating properties and enzyme cost 23
Extracted Structural features 24
Grouping of amino acids 25
Evaluated experimental data sets and databases 25
Flux and kcat calculations 25
Enrichment analysis 26
Correlation and statistical analysis 26
Network clustering 27
Machine learning 28
Structural representation 29
Evolution rate (dN/dS) calculations 29

Supplemental Tables

Table	Notes
S1.1	Lists of selected species and source of sequences for each species.
S1.2	List of structures that failed to calculate.
S1.3	Average pLDDT and percentage of structures calculated for each orthogroup.
S1.4	Orthogroup, species, UniProt ID, Length, Cluster and pLDDT for structures that we use for analysis. Highlight if structure was predicted during the course of the study or extracted from AlphaFold DB.
S1.5	Table of refined clusters generated from original orthogroups based on TM-align scores. Includes list of *S.cerevisiae* genes in each cluster.
S1.6	Table including TM-scores of all refined orthogroups.
S1.7	Selected metrics and calculated values for each structural orthogroups, like CR, MR, Mapped length, Amino acid content and protein abundances.
S5.1	General descriptions on the network clusters. To remove artefacts, clusters have to have at least a size of 5. The median value as well as the interquartile range (IQR) are presented for both binding site definitions.
S6.1	Key residues highlighted in Figure ED9a,b for the orthogroup containing *S. cerevisiae* Pox1p across different phylogenetic orders.
S7.1	Evaluated experimental datasets and databases.

Supplementary Notes

Note 1: Choice of *Saccharomycotina* yeasts for addressing metabolic evolution

The yeast subphylum *Saccharomycotina* shared a common ancestor around 400 Million years ago (MYA). Unlike bacteria, however, there is much less horizontal gene transfer which would complicate comparisons across different orthogroups of proteins. At the same time, they are still characterised by a remarkable metabolic diversity, including gene losses preventing growth on specific substrates [1,2], a major whole-genome hybridization that corresponds with the emergence of the ability to ferment in the presence of oxygen (Crabtree effect) [3–5], and limited horizontal gene transfers from other yeast and bacteria that confer new metabolic capabilities [6–9]. The clade has been comprehensively sequenced and characterised at the molecular and metabolic level [1,2,10–14], and includes the most prevalent human fungal pathogen *Candida albicans,* and several industrially important yeast species (*Kluyveromyces marxianus, Komatagella pastoris, Yarrowia lipolytica)* as well as the model single celled eukaryote and food and beverage industry workhorse, *Saccharomyces cerevisiae.* Previous work has integrated phenotypic and genomic evidence to understand the evolution of metabolism in the yeast subphylum [1,2], including reconstruction of individual genome scale metabolic models for hundreds of species [15,16].

Note 2: Limitations of AlphaFold2 in relation to our study

It has been shown that the quality of AlphaFold2 predictions can vary depending on the predicted protein. Although in many cases there is a good agreement with experimental data, in some cases, inaccuracies on the global or local level have been observed. Examples are domain displacements or inaccurate side chain orientations [17]. We observe a few rare instances of domain displacement in our data, however, since we observe a median mapping ratio of 87.4% and we detected this behaviour only sparsely, it should not strongly influence our analysis. Since our mapping and analysis protocols are based mainly on the backbone configuration, inaccuracies in the side chains should also not influence the presented results. Another issue that arises is AlphaFold2's tendency to wrongly predict random coil regions as helical (2.02%) more than extended (0.26%)[18]. If we assume 2.28% wrongly predicted random coil regions, this would result in an estimated maximal error of 1.0% (based on the relative quantity of random coils), less than

the mean difference of 9.7% we observe between helical and extended structures (Figure 5d). Thus, we can neglect this error in our analysis.

In this manuscript we assigned the binding site using evidence from crystal structures in the PDB. This was done because in Alphafold2 structures do not explicitly include substrates, cofactors, other protein chains and other coordinating subunits. However, since AlphaFold2 is a deep-learning framework that utilises existing structures which contain these additional molecules, it implicitly incorporates information about their presence without retaining the nature and extent to which that information is included [19]. The very recent publication of Alphafold3 allows the inclusion of such interactions and could be incorporated in future work [20]. Additionally, Alphafold2 does not accurately capture structural information from (intrinsically) disordered domains [21] because the dynamical properties of the protein are not included in its training. Dynamic disordered domains, although present, [22–24] are less common in metabolic enzymes than they are in some other classes of proteins [25,26], so by focusing on metabolic proteins, we avoid this issue to some extent.

Another limitation that arises from the dynamic nature of protein structures is that, in the case of complex formation, different metastable conformations may be formed, which our monomeric static structures would not account for. One possible method to account for this would be to predict structures in the context of their biological complexes using approaches like AlphaFold Multimer [27] or AlphaFold3 [20]. However, the high mapping ratios that we achieve within our orthogroups (Figure 1d) indicate that structural differences between complex-bound and unbound proteins only add a minor bias to our analysis. To assess this, we aligned our predicted structures of *Saccharomyces cerevisiae* to 553 experimentally determined homo- and heteromeric complex structures from RCSB PDB based on 106 reference structures, obtaining a mean mapping ratio of 0.91 (Figure ED7c). This analysis shows that since AlphaFold2 is trained on experimental data it captures protein structures in complexes as well, at least to a certain amount. Also comparing the distributions of structures that are in complex (ComplexomeDB) with the remaining structures, we see no significant difference in the mapping ratio (Figure ED7d).

Note 3: Further details on specific hypotheses generated from structural analysis.

Note 3.1: Xylose pathway specialization

When we separated orthologs based on whether they were from species that exhibited various phenotypes (Fig S2.1A), we observed the most significant difference in CR relative to the reference structure for the xylose utilization phenotype. To further explore this, we focussed on orthogroups with exceptional (top 10%) of differences in CR between species that can or cannot use D-xylose in greater detail (Figure 2b). Several of these orthogroups were in the pathways of xylose metabolism and downstream pathways [28]. The orthogroup containing transketolase (Tkl1p and Tkl2p in *S. cerevisiae*), as well as 3 out of 4 orthogroups from the thiamine biosynthetic pathway were included in this set of proteins. In yeast that use xylose, transketolase integrates the immediate downstream product xylulose 5-phosphate into central metabolism via the pentose phosphate pathway. Thiamine is an essential cofactor for transketolases (Tkl1,2p) (Muller et al 1993), and [29] observed improved growth on xylose following deletion of Thi2p in engineered *S. cerevisiae*. Another possible connection between enzyme conservation and xylose utilisation in our data involves the metabolic shift between aerobic and anaerobic growth. Acetyl phosphate (AcP) is a major breakdown product of xylose when it is metabolized via phosphoketolase in some yeast [30]. Though the metabolism of AcP has not been well studied in yeast, some eukaryotes are able to convert AcP into acetyl-CoA via acetyl kinase and acetyl-CoA synthase (ACS) [31]. ACS has two paralogs in several of the species we studied, and in *S. cerevisiae* the paralogs are Acs1p and Acs2p, which are expressed in aerobic and anaerobic conditions respectively. In the species that utilize xylose and contain both paralogs (*C. tropicalis, O. parapolymorpha, P. tannophilus* and *G. candidum*), one protein is more similar to the *S. cerevisiae* Acs2p (CR = 0.68, anaerobic) than to Acs1p (CR = 0.59, aerobic), however, the other protein is not more similar to *S. cerevisiae* Acs1p, but is similarly divergent from both both *S. cerevisiae* ACS paralogs (Acs1p, CR = 0.63 versus Acs2p, CR = 0.60)(Figure ED2d,e). This indicates that Acs1p, expressed more in aerobic conditions in *S. cerevisiae,* may specialize based on whether it is in a xylose utilizing species. Furthermore, [32] observed an increase of expression for Acs2p and a decrease for Acs1p in an engineered strain of *S. cerevisiae* evolved for increased growth on xylose. They also observed a decrease in ETC protein expression, while we see that 6 out of 22 ETC proteins have a large difference in CR between xylose utilizers and non-utilizers. Taken together, this indicates that between *S. cerevisiae* and the species that can utilize D-xylose, there may have been a

divergence between the aerobically expressed Acs1p gene and its orthologs, as well as changes in the ETC, the thiamine pathway and the thiamine dependent transketolase that may influence the way that these species process xylose.

Note 3.2: Hsu1p/Str2p structural divergence

OG1390 contains three paralogous proteins in *S. cerevisiae*, Hsu1p, Str2p, and YML082Wp in *S. cerevisiae*. Str2p and Hsu1p do not rescue one another in genetics experiments and are thought to perform different but similar chemical reactions (Str2p: Cystathionine Gamma Synthase (EC 4.2.99.9), Hsu1p: Homocysteine Synthase (EC 2.5.1.49), and Cysteine Synthase (EC 2.5.1.47)) [33–35]. All of the proteins in the orthogroups contain two domains, CATH 3.40.640 and 3.90.1150, and many contain a third domain which varies from protein to protein and often does not map to a CATH ID. Based on visual inspection and alignment with a crystal structure from Met17p (8OVH), a more distantly related protein in a different orthogroup which shares the Homocysteine Synthase reaction with Hsu1 (EC 2.5.1.49), the structure and specific residues of the reaction site are largely conserved with one or two intriguing exceptions. The more variable domain is located in a location where it would disrupt homo-tetramerization observed in Met17p and in an orientation that might influence access to the binding site. We speculate that this domain and some of the conserved changes near the active site would determine specificity of the reaction.

Note 4: Description of cost metrics

To estimate cost, we made use of several established frameworks, which assign numerical values to indicate the share of the cell's resources required to produce amino acids, and which can be summed to estimate the cost of a protein. The cost metrics that we used were based on three main principles and have varying degrees of species dependence.

The first is molecular weight [36], which does not change between species.

The second principle estimates the biosynthetic cost based on the energy required to make each amino acid using the nearly universal central metabolic pathways that produce them. These are actually based primarily on data from *E. coli* and take into account either the number of steps or the energy (phosphate bonds and equivalents) required to synthesize the amino acids. Cost metrics using this principle include Craig and Weber Steps, Craig and Weber Energy Equivalents [37], Akashi and Gojobori Energy Equivalents [38], and Wagner Respiratory and Fermentative Protein Synthesis costs [39,40]. As these are based on core metabolic pathways and reactions contained in

this subclade and in most living organisms, they are not likely to be sensitive to the difference between species.

The final two metrics are based on whole scale metabolic models in *S. cerevisiae*, and include Glucose Cost [39,41] and Protein Cost [41]. In both of these frameworks, the first step is to increase the requirement for a given amino acid in the model with a small perturbation while holding growth rate steady and measuring how much the model changes. For Glucose Cost, the increase in the amount of glucose required in the model is used. For Protein Cost, an estimate of the increased metabolic cost of producing the enzymes required to make the desired metabolite is used. The costs assigned to each amino acid using these methods was shown to be inversely correlated with overall amino acid abundance in budding yeast [41] which we interpret as evidence of these costs shaping amino acid content over time, and Protein Cost shows the strongest correlation.

In order to test for the possibility that species specific differences in metabolic networks, and the resultant differences in synthesis costs could confound our results, we have compared network based cost metrics with those that are independent of the species-specific metabolic networks (i.e. those which are based on molecular weight or energy equivalents within core metabolic pathways common to all species), with the metrics that are based on species specific metabolic networks. We obtained similar results (Figures 4a,b), and can thus conclude that our results are robust to divergence of the metabolic network structure, and species-specific differences in cost.

Note 5: Observations that require known structures

In this work, we aimed to investigate structural changes that might affect evolution on a large scale which might not be visible when analysing protein sequences, or the smaller set of known experimental structures alone. We found that surface residues were less conserved than interior residues. Reasons for this might be multifold: increased steric hindrance due to close packing of the protein, stabilising interactions, protein folding and solubility. In addition, surface residues might be more prone to mutational drift if they don't fulfil a functioning, coordinating or stabilising purpose [42]. Also for extended structures and random coils a higher conservation than for helical regions was observed. This may be partially explained by a higher number of interactions between the residues of the extended structures and random coils than within helical structures. In the case of random coil structures, we also only considered residues that could be mapped to the respective reference structure and thus seem to have, at least in the prediction, a defined spatial arrangement. These mapped regions of random coils were neither more conserved than other

mapped residues (Figure ED7a) nor less conserved than other secondary structural elements (Figure ED6d), though tight turns, a subset of the random coils, were more conserved (Figure ED7a).

The structural information that we had for our orthogroups also allowed us to better understand relationships between conservation and relative amino acid content, a sequence-based factor. We were able to further analyse these relationships in the context of different structural features to determine how those features contributed to the relationship. We find, for instance, that the correlation between alanine content and conservation is more specific to helical regions, while the correlation between glycine content and conservation is more specific to random coil regions. For alanine it might be explained by its helix formation properties [43] while for glycine it might reflect the increased backbone flexibility due to the lack of a side chain [44]. To analyze the impact we investigated tight turns [45] in our structures, which can profit from an increased backbone flexibility. Indeed, for glycine, we see an increased and for alanine a decreased content in tight turns including a dependency on the length of the turns (Figures ED7e,f). Furthermore, for glycine, a higher conservation than expected was observed in tight turns (Figure ED7g). However, since glycine is more conserved than other amino acids and tight turns are also more conserved than the overall structure (Figure ED7a,g) it is hard to capture how much this effect is due to the higher glycine content in tight turns. We also observed differences for core and surface residues. The fact that the correlation between CR and amino acid content is stronger for alanine within inner residues and for glycine within outer residues might be due to the more hydrophobic nature of alanine compared to glycine [46]. This observation is consistent with a notion of highly conserved proteins optimising for cost in external residues by replacing more expensive amino acids with less expensive glycines when possible, and doing the same with alanines for internal residues. We also see a surprising anticorrelation between leucine content and overall conservation, which is more prevalent when considering helical and coil regions than when considering extended regions and might be a direction for future research.

Also for cysteine an anticorrelation with CR is observed only for outer residues, likely since it is low in overall abundance (Figure ED5b), and cysteine typically has specific dedicated functions in the protein related to its reactive nature, such as catalytic activity or stabilisation via disulfide bridges. In the human proteome, for example, a recent study observed that cysteine surface accessibility was related to the dedicated function of the cysteine [47]. One way of understanding this observation is to consider that cysteine on the outer residues of a protein may cause an

increased burden on the cell for expressing the protein, thus driving a negative correlation with conservation as selection tends to minimise that burden. This may be due to selection against spontaneous, unintended reactions of surface-exposed cysteines in more conserved proteins [48]. For other amino acids, such as proline, no significant correlation was observed, and, for proline, a median correlation around zero was obtained. This could possibly be related to the fact that proline limits not only its own backbone flexibility, but also that of its preceding amino acid [49] and thus might be important for a structure's overall spatial arrangement.

Note 6: Investigating signals of evolutionary selection in enzyme evolution

Note 6.1: Higher degree of evolutionary selection in some orthogroups

We addressed whether an increase in diversity is explained by evolutionary selection. We focused on the orthogroups that contain the most variable enzyme structures and asked to which degree their increased diversity represents drift or positive selection by comparing the diversity detected within the orthogroups with dN/dS values, that in evolutionary genomics are used to distinguish selective neutrality (dN/dS of ~1) from positive (> 1) and purifying selection (< 1) [50,51]. We calculated dN/dS ratios based on all-vs-all alignments to initially calculate a single rate per orthogroup (M0 model) to assess the extent of this [52,53]. Although we obtain an average dN/dS of < 1, indicating overall purifying selection occurred for each orthogroup, we report substantial differences depending on the enzymes. Relatively high values of dN/dS may indicate orthogroups with higher proportions of residues under neutral drift (dN/dS = 1) or positive selection (dN/dS > 1). As we cover a large evolutionary timespan (400 my), we neglected orthogroups with an average dS value of > 3 to avoid skewing of our dN/dS calculations, at the expense of removing some low conserved enzymes, enriched for GO terms related to glycosylation (Figures ED8a-c). For the remaining orthogroups, we performed enrichment analysis of the dN/dS values (Figures ED8d). For orthogroups in the first quartile of dN/dS values, glucose fermentation and ETC pathways were enriched, as well as the GO slim terms “oxidoreductase activity”; “monocarboxylic acid metabolic process”; the molecular function term “transmembrane transporter activity”; and the cellular compartment terms “membrane”, “mitochondrion”, and “mitochondrial envelope”. No terms or pathways were significantly enriched in the orthogroups of the last quartile of dN/dS values. Although no enzyme class was significantly enriched, there were differences at the level

of individual enzymes with specific oxidoreductases and isomerases, as well as specific enzymes of the the GO term “lipid metabolic process”, having high dN/dS values (Figures ED8e,f).

To investigate the reduction of purifying selection in some orthogroups in more detail, we obtained dN/dS estimates at the amino-acid level (site-model) for those orthogroups which had dS<3 in the M0 model [54–56]. Most orthogroups were better represented with a two-class (Nearly Neutral) model (Chi2 test threshold of 6.63) neglecting positive selection. Overall we observed a lower probability of neutral drift for highly conserved orthogroups (Figure ED8g) and, breaking the analysis down by structural features, we observed a higher probability of neutral drift in surface residues (Figure 5e). Three orthogroups, including those containing *S. cerevisiae* glycolytic enzymes glyceraldehyde-3-phosphate dehydrogenase (GAPDH) (Tdh1/2/3p), Triosephosphate isomerase, Tpi1p, and Fumarate hydratase Fum1p, were better represented with a three class model in which each residue could also experience positive selection as well as purifying selection or neutral drift. In addition to these enzymes, our interest was also triggered by the orthogroups that were highly diverse (CR ≤ 0.6) yet had higher dN/dS values (> 0.06), indicating a higher proportion of residues under neutral drift, or positive selection in specific branches, rather than purifying selection. This group included several proteins of the ETC, such as complex III (in 6 of the 9 orthogroups) and complex IV (in 4 of the 8 orthogroups) as well as the fatty acyl coA oxidase Pox1p that is part of the fatty acid β-oxidation pathway (Figure ED8h).

Note 6.2: Evolutionary selection affects structural elements differently

To analyse where signals of positive selections are located in the protein structure within specific branches, we applied a branch-site model to obtain dN/dS estimates at the amino-acid level [53,56–58]. Notably, these regions were identified in diverse structural elements.

In the Pox1p orthogroup’s protein tree, we identified 15 branches showing evidence of positive selection (Figure ED9a). Within the branch encompassing *S. cerevisiae* and *Wickerhamomyces anomalous*, 15 residues showed signatures of positive selection, including residues aligning with Lys580, and Gln653 in *S. cerevisiae*, which are in close contact at the homodimeric interface. We

report that the basic Lys580 appeared to physically interact with the acidic Asp631 across the interface in this branch, stabilized by Gln653 and Tyr385 (Figure ED9b). In contrast, outside of this branch, these interactions across the homodimer interface were absent, for instance in the branch containing *Y. lipolytica* ylAOX1 (Figures ED9a,b, Table S6.1). We speculated that these residues may have been selected in the common ancestor of *S. cerevisiae* and *W. anomalus* for stronger interactions at the homodimeric PPI site, as the interaction occurs twice on the surface, which could enhance fitness even more.

In contrast, many positively selected residues in GAPDH were located primarily on surface-exposed residues, but none on homomultimeric PPI sites. Furthermore, we detect one positively selected lysine in the branch encompassing proteins from *P. tannophilus* to *S. cerevisiae* which appears to influence the catalytic site. Between *S. cerevisiae* and *C. jadinii* it is conserved alongside a coordinating glutamate and is close to the binding pocket of the substrate and the NAD cofactor and might affect the substrate binding. Outside of this clade the two residues both tend to change to threonine which is smaller and thus more distant from the active site (Figures ED9c,d).

For the ETC, a large supercomplex that spans the membrane which separates the mitochondrial matrix from the intermembrane space, we obtained up to 11 branches with evidence of positive selection with up to 30 positively selected residues per branch for each orthogroup. To investigate the PPI sites at which the most positively selected residues are present, we analyzed the ubiquinol cytochrome c reductase/cytochrome c oxidase supercomplex ($CIII_2$-CIV_2 mitochondrial respiratory supercomplex, PDB: 6HU9 [59] and detected several instances, where a complex member showed positively selected residues at PPI site. Indeed, of the 157 unique positively selected residues identified, 96 were on PPI sites. Within a complex, the homo-dimeric Qcr2p-Qcr2p and the Qcr2p-Qcr1p interfaces had the most positively selected residues, and between complexes, the connection interface Cox5Ap-Qcr1p had the most (Figure ED9e-g). Of note, the homodimeric Qcr2p-Qcr2p interface contained five positively selected residues in the branches containing orthologs from *S. cerevisiae* to *W. anomalus* and to *A. rubescens* respectively that form a cluster on the interface which amplifies any effects due to the dimeric characteristic of the interface (Figures ED9e,g). The orthogroups containing mitochondrial encoded ETC members were underrepresented as they did not pass our inclusion criteria. Nonetheless, there were four positively selected points of contact between the nuclear encoded Cox7p and the mitochondrially encoded Cox3p, and five positively selected points of contact between the nuclear encoded

Cox9p and the mitochondrially encoded Cox2p (Figure ED9g). We hypothesised that positive selection is stronger for these nuclear encoded proteins, as the mitochondrial genome has been shown to have a higher mutation rate than the nuclear genome in more recently diverged branches of budding yeast (Christinaki et al., 2022), which could also explain their high structural diversity. To assess this, we compared the total number of amino acids present along an axis of symmetry perpendicular to the membrane to the number of amino acids along that axis that showed positive selection. In the central part of this axis, where there was a higher proportion of mitochondrially encoded proteins, there was a greater number of positively selected residues, although the difference in density distributions of all residues versus that of positively selected residues was not significant (two-sided, two-sample Kolmogorov-Smirnov test, $p < 1e-1$).

Thus, positive selection affects structural elements differently and can play different roles in enzymes, affecting complex formation to substrate interaction. Identifying residues under positive selection can thus act as a hypothesis generating starting point for future studies.

Methods

Orthogroup and Species Selection

We selected a range of 26 sequenced budding yeast species that span the diversity of the *Saccharomycotina* subphylum, as well as the model fission yeast *Schizosaccharomyces pombe,* as an outgroup to root the tree. Nine of the species were selected, including the model budding yeast, *S. cerevisiae*, were identified in [14] as a phylogenetically diverse set of yeast species representing the NCYC strain collection. We also chose the medically relevant commensal and pathogenic yeast, *C. albicans* among 15 other species chosen to represent all the major clades of the subphylum as described in [2] (Figure 1a, Table S1.1).

Orthogroups were assigned as per orthoMCL clusters from [2]. In order to select orthogroups for the study, we began with genes assigned to yeast pathways per a yeastmine query mapping pathways to genes executed on 07 Oct 2021 [60]. We used 517 of the 555 genes in the pathway database [61]. The missing 38 genes came from 51 pathways not included in our yeastmine query due to a data error that we reported to SGD and which has been fixed. From these 517 genes we

obtained 445 orthogroups as some orthogroups contained paralogs of some pathway genes. From those orthogroups, we kept 426 orthogroups that had genes present in at least 40% of the species. Those 426 orthogroups contained 534 genes in *S. cerevisiae* and, of those, 499 genes (90% of the 555 genes in the pathway database) were associated with 224 of the 229 (98%) pathways in the yeast pathways database.

Structural predictions using AlphaFold2

We downloaded structures for *S. cerevisiae*, *C. albicans* and *S. pombe* from the AlphaFold Protein Structure Database [19] on 27 Apr 2022 [*S. cerevisiae* (n = 534), *S. pombe* (n = 375) and *C. albicans* (n = 392)]. For the other species we predicted protein structures based on sequences using Alphafold2. We attempted to obtain protein sequences from uniprot for the 9 species identified by [14] and for which we had proteomics data. As the genes name from [2] and that from uniprot did not correspond, we matched proteins by calculating pairwise protein similarity between each gene identified in [2] and every gene in the uniprot proteome using the biopython function pairwise2.align.globalms with the following parameters: match_points = 1, mismatch_points = -1, gap_open = -.5, gap_extension = -0.1, and penalize_end_gaps=True. Where there was a similarity score above 75, or where there was a similarity score above 60 with a difference of 8 or greater above the next highest similarity score, we used the uniprot protein sequence. Where these conditions were not met, we used the protein sequence from [2]. For species not studied in [14] we used protein sequences from [2].

We provided 10,545 sequences as input to Alphafold2 version 2.0.1, installed on the Berzelius computing infrastructure and using the default settings with the full BFD database. The structure with the best pLDDT-score of the 5 output models was used as our final structure. We were unable to calculate structures for 577 sequences, and the distribution of these sequences was skewed in terms of sequence length, species, and orthogroup (Tables S1.2-3). For the sequences for which we couldn't calculate structures, 20.8% had a length greater than 1800 residues, compared to only 0.6% of the sequences for which we could calculate structures. This length dependence was apparent in the bias in the unpredicted structures towards specific orthogroups. 29.3% of uncalculated structures were in 7 orthogroups for which structures could not be generated in over half of the sequences of the orthogroup. This included two orthogroups for which no structures could be generated for any of the sequences (27 sequences from OG1710 which includes the *S. cerevisiae* proteins Hfa1p and Acc1p, and 24 sequences from OG1869 which includes the *S.*

cerevisiae gene Fox2p). For these 7 orthogroups, 55.6% of their sequences were longer than 1,800 residues (Table S1.2).

There were also more unpredicted structures in *Hanseniaspora osmophelia*, *Zygosaccharomyces rouxii*, and *Yarrowia lypolitica* relative to other species (163, 131, and 48 unpredicted structures respectively), comprising 59.3% of the unpredicted structures. Length did not appear to be a strong factor in preventing the prediction of these sequences, as only 5.0% of them had length greater than 1,800. In sum of the 577 structures we could not calculate, 88.4% were either from one of these three species, in one of the 7 most challenging orthogroups, longer than 1,800 residues, or a combination of those factors. Following structure prediction we combined our predicted structures with those predicted in the AlphafoldDB from our model organisms and carried 11,269 structures from 424 orthogroups forward for further analysis.

For the pLDDT, especially for the N-terminal region low values were obtained. One reason might be that the annotation of translational start sites can be difficult to predict from genomic sequences, and alternative canonical start codons as well as alternative non-canonical start codons can be used for various genes [62]. Thus it is possible that for some genes the annotated N-terminal region is not expressed. Also both N-terminal and C-terminal regions of a protein often contain localization sequences, regulatory domains, or domains that modify protein-protein interaction [63,64]. These are often unstructured and may be cleaved and degraded, therefore these regions may not be selected for structural stability.

Multiple structure alignment and Orthogroup refinement

For each of the orthogroups, multiple structure alignments were carried out initially on the 424 orthogroups by aligning the structures in each orthogroup to their respective *S. cerevisiae* reference structure using the *matchmaker()* function provided by *ChimeraX 1.2.5* [65–68] using default parameters, the Needleman-Wunsch algorithm [69] and CA-matching. UCSF ChimeraX, is developed by the Resource for Biocomputing, Visualization, and Informatics at the University of California, San Francisco, with support from National Institutes of Health R01-GM129325 and the Office of Cyber Infrastructure and Computational Biology, National Institute of Allergy and Infectious Diseases. We noticed that some orthogroups had proteins that clustered together, so we refined our original sequence based algorithms based on structural similarity.

To do this refinement, we began with US-align which is an extended version of TM-align [70,71] for bidirectional structure alignment. We chose to use TM-score instead of RMSD because TM-score better captures global similarity by weighing closer distances between aligned atoms more heavily than larger distances, and preventing a single poorly aligned region from overly influencing the overall score. This can be seen by inspecting the equations for each. The equation for RMSD is:

$$RMSD = min\left(\sqrt{\frac{1}{N}\sum_{i=1}^{N} (d_i)}\right) \quad (1)$$

where N is the length of the two aligned proteins and d_i is the distance between aligned C_α atoms. The equation for TM-score is:

$$TMscore = max\left(\frac{1}{N_T}\sum_{i=1}^{N_S} \frac{1}{1+\left(\frac{d_i}{d_0(N_T)}\right)^2}\right) \quad (2)$$

where

N_S is the length of the residues that appear in both the Target and the template structure, N_T is the length of the residues in the Template structure, d_i is the distance between aligned C_α atoms and

$$d_0(N_T) = \sqrt[3]{N_T - 15} - 1.8 \quad (3)$$

is a scaling factor for distances based on the overall length of the template structure.

The fact that the d_i are in the denominator in TM-score (equation 2) means that smaller distances are weighted more than larger distances in the calculation unlike in RMSD (equation1) where they are weighted equally. Additionally, TM-score contains a normalizing factor (equation 3) that makes it independent of the overall protein lengths and equal to approximately 0.17 for two random proteins. This is different for RMSD, for which two random large proteins would tend to have a worse RMSD than two random small proteins.

US-align was used to align each predicted protein structure to every other predicted structure that was assigned in the same orthogroup. Following this, subclusters within each of the 424 original orthogroups were generated using hierarchical clustering using scipy.cluster.hierarchy.linkage with default parameters. There were 12 orthogroups that contained at least one protein that did not cluster with any other protein of the orthogroup based on a linkage threshold of 0.2 (scipy.cluster.hierarchy.dendrogram labelled as C0 with color_threshold=0.2. This subclustering resulted in 29 orthogroups being split into two subclusters, except in the case of OG2228 containing Psd2p from S. cerevisiae which was split

into three subclusters. The subclusteried orthogroups had an improved average TM score after clustering (0.773) compared to before clustering (0.710) (Table S1.5). This resulted in a total of 454 refined structural orthogroups uniquely identified by the orthogroup and a reference structure within that cluster (Table S1.6)

Structure alignments for the refined structural orthogroups were then extracted as subsets of the original reference-based structure alignments and used as the basis for calculations of mapping ratios, conservation ratios and structural and metabolic analysis. For these calculations 25 refined structural orthogroups were discarded that had no *S. cerevisiae* reference structure assigned, leaving 429 refined orthogroups. From 531 remaining reference structures in *S. cer.* two structures couldn't be assigned to any cluster and were therefore neglected for further analysis resulting in a final number of 529 reference structures.

For calculation of dN/dS as well as creating phylogenetic trees for each orthogroup, new multiple structure alignments were generated for each refined structural orthogroup without mapping to the reference structure by running US-align on the refined structural orthogroups with the option -mm 4.

For these alignments we then filtered out 135 structures whose sequences were less than 80% of the median length of their structural orthogroup, and then filtered out 22 structural orthogroups that had 3 or fewer sequences. Each refined orthogroup was then assigned a reference structure for naming purposes, which was from *S. cerevisiae* in all but 7 of the refined orthogroups. This left 432 refined orthogroups that were carried forward for further analysis. Genes were designated as paralogs if they were from the same species in the same refined orthogroup.

Multiple sequence alignments and phylogenetic trees

Multiple sequence alignments (MSAs) were generated for the 432 refined orthogroups for which short sequences and orthogroups with 3 or fewer structures were removed. Structure informed alignments (US-Align) versions were generated with the US-Align program with the option -mm4. To generate phylogenetic trees for each MSA alignment were first trimmed using clipkit version 1.4.1 with default parameters [72]. IQtree version 2.1.4-beta was then run on each alignment using default parameters that allow different evolutionary models to be identified and used for each

sequence. The options –bb 1000 and -alrt 1000 to evaluate the quality of each tree [73]. Illustrations of multiple sequence alignments were performed using Jalview V2.11.4.1[74].

Metabolic network visualisation

Visualisation of the yeast metabolic pathways was performed using iPath3 [75]. Of the 529 *S. cerevisiae* structures from 429 orthogroups we analysed, 448 were present in the iPath3 database metabolic network, covering 62.5% of the 717 *S. cerevisiae* uniprot IDs present in the iPath3 metabolic network. Overall, the iPath3 metabolic network contained 243 more proteins than the yeast pathways database which we used to identify enzymes and orthogroups of interest. Colour and width of each element in the metabolic map was determined by the average conservation scores for the proteins assigned to that element in iPath 3 for the full KEGG metabolic map (01100) subsetting on *S. cerevisiae* (sce).

Phenotype selection

Data for 72 different growth phenotypes for various *Saccharomycotina* species was taken from [16], which assembled data originally collected in [10]. We filtered out 46 conditions which had data from fewer than 5 species that can grow and fewer than 5 species that can not grow under the respective condition. We further filtered 4 conditions that had missing data in more than 4 species, and the condition of growth at 37 °C as it was the only remaining condition that was not a change in carbon source, leaving phenotypes for growth in 21 carbon sources. Where the data stated that there was variable growth in a species for a particular phenotype, that species was removed from the analysis for that phenotype. For the analysis we temporarily created two subgroups, one containing all species that can and one containing all species that can not utilize a specific carbon source. The measurements for fermentation were published in (Kurtzmann et al 2011, see pgs. 100-101), and strains were tested for the production of Carbon Dioxide using inverted Durham tubes over a period of up to 28 days at 25-28 °C. Fermentation of sugars was verified by the presence of a bromothymol blue indicator which changes from green to yellow to indicate acidification of the media.

Determining protein abundance using mass spectrometry

Cultivation

Strains were streaked on a minimal nutrient medium (consisting of yeast nitrogen base with 1% glucose) and 2% agar plates and incubated at 25°C for 48 h. Single colonies were selected, inoculated into 60 ml of yeast nitrogen base with 1% glucose medium (300 ml flask total volume) and incubated for 24 h at 25°C with shaking at 200 r.p.m. (Sartorius Certomat IS Shaker). Subsequently, optical density at 600 nm (OD600) was recorded and 20 ml of culture were centrifuged at 1,500 x g for 5 minutes at RT (Eppendorf Centrifuge 5810 R). After centrifugation, the supernatant was discarded and the cell pellet was resuspended in 20 ml of the same fresh medium (flask size was adjusted to 100 ml to keep the same liquid-to-air ratio of pre-cultures). Cultures were placed back into the incubator for shaking with the same settings (25°C, 200 r.p.m.). After 6 h of incubation, corresponding to mid-log phase,2 ml of culture were sampled into screw cap tubes. A small volume of 50 µl was removed for OD600 measurement while the remaining sample was immediately centrifuged at 21,000g (Thermo Scientific Heraeus Fresco 21 Microcentrifuge) for 1 minute at 4°C, then the supernatant was carefully removed by inversion and the cell pellet was flash frozen with liquid nitrogen. Finally, ~130 µg of pre-aliquoted glass beads (425-600 µm) were added to each sample tube with a small funnel and frozen at -80°C until further processing.

Processing QC samples were generated by cultivating the prototrophic lab strain *S. cerevisiae* BY4741 HLUMki, an auxotrophy-repaired derivative of BY4741 [76]. in rich Yeast Peptone Dextrose (YPD) medium. A single flask of 400 ml YPD was inoculated with BY4741 HLUMki directly from the cryostock. The culture was incubated for 20 h at 30°C with shaking at 200 r.p.m. (Sartorius Certomat IS Shaker), then distributed into aliquots of 4 x 1e+08 cells. Cells were harvested by centrifugation at 21,000g (Thermo Scientific Heraeus Fresco 21 Microcentrifuge) for 1 minute at 4°C, then the supernatant was removed and the cell pellet was frozen at -80°C until further processing.

Cell lysis

Frozen pellets were topped with 200 µl of lysis buffer (7M urea, 0.1M ammonium bicarbonate) and lysed in a bead-beater (SPEX, SamplePrep 1600 MiniG) for 5 minutes at maximum speed (1,500 r.p.m.). The procedure was performed three times for a total of 15 minutes of beating per

sample and each cycle was followed by 5 minutes of cooling down on ice to avoid sample overheating.

After 1 min of centrifugation at 15,000 r.p.m. (Thermo Scientific Heraeus Fresco 21 Microcentrifuge) at 4°C to clear the lysate, protein concentration was measured by Pierce 660nm Protein Assay (ThermoFisher). The volume corresponding to 100 µg protein was transferred to a 96-well 2 ml deep-well plate (Eppendorf) and total volume was adjusted to 200 µl with lysis buffer.

Sample preparation for mass spectrometry

Sample preparation was adapted from [77]. Samples were treated by addition of 20 µl of 55-mM DL-dithiothreitol (final concentration 5 mM). The plate was mixed for 2 minutes at 1,000 r.p.m., and incubated for 1 h at 30 °C. After incubation, samples were cooled on ice for 5 minutes. Subsequently, 20 µl of 120 mM iodoacetamide was added (final concentration 10 mM), then mixed for 2 minutes at 1,000 r.p.m., and incubated for 30 min in the dark at room temperature. Subsequently, 460µl of 100-mM ammonium bicarbonate was added. Samples were mixed for 90 seconds at 1,000 r.p.m., and an aliquot of 500 µl was transferred to pre-filled trypsin/LysC plates (Waters, 96-well plate 700 µl round, 4 µg of trypsin/LysC). After incubation of the samples for 17 h at 37 °C with shaking at 750 r.p.m. (Benchmark Scientific, Incu-Mixer Microplate vortexer), trypsin/LysC were deactivated by addition of 17 µl of 30% formic acid (final concentration 1%). The digestion mixtures were cleaned using C18 96-well plates (96-Well BioPureSPN, PROTO 300 C18, 35-350µg max capacity, The Nest Group, no. HNS S18V-L). For solid-phase extraction, 1-min centrifugation steps at the described speeds (Eppendorf Centrifuge 5810 R) were applied to force liquids through the stationary phase. A liquid handler (Beckmann Coulter Biomek i7) was used to pipette the liquids onto the material. The plates were conditioned with methanol (200 µl, centrifuged at 50g), washed twice with 50% acetonitrile (ACN, 200 µl, centrifuged at 150g and flow-through discarded) and equilibrated three times with 0.1% formic acid (200 µl, centrifuged at 150g, respectively, and flow-through discarded). Then, 500 µl of digested samples was loaded (centrifuged at 550g) and washed two times with 0.1% formic acid (200 µl, centrifuged at 150g). After the last washing step, the plates were centrifuged once more at 200g before elution of peptides in three steps, each with 110 µl of 50% ACN (200 g), into a collection plate (Waters, 96-well plate 700 µl, round). The collected material was completely dried on a vacuum concentrator (Thermo Scientific, Savant SpeedVac SPD300) and redissolved in 90 µl 0.1% formic acid by mixing for 5 minutes at 1,000 rpm before transfer to a 96-well plate (700 µl round, Waters, no. 186005837). Peptide concentration was measured using Pierce Quantitative Peptide Assay (ThermoFisher Scientific). All shaking was performed with a thermomixer (Eppendorf

Thermomixer C) and, for incubation, a Benchmark Scientific, Incu-Mixer Microplate vortexer was used.

Liquid chromatography mass spectrometry

Samples were analysed on a Bruker timsTOF Pro mass spectrometer, coupled to a Dionex Ultimate 3000 µsystem (Thermo Fisher Scientific). Prior to MS analysis, 1µg peptides were chromatographically separated with a 30 min gradient on a Waters HSS T3 column (300um x 150mm, 1.8um) heated to 40°C, using a flow rate of 5 µl /min where mobile phase A & B are 0.1% formic acid in water and 0.1% formic acid in ACN, respectively. The active gradient increases from 2% to 40% B in 30min.

For diaPASEF acquisition, the electrospray source (Bruker Apollo II source, Bruker Daltonics) was operated at 4500 V of capillary voltage, 5.0 l/min of drying gas and 200 C° drying temperature The dia-PASEF windows scheme was as followed: we sampled an ion mobility range from 1/K0 = 0.6 to 1.60 Vs/cm2 using equal ion accumulation and ramp times in the dual TIMS analyzer of 100 ms, each cycle times of 0.5 s. The collision energy was lowered as a function of increasing ion mobility from 59 eV at 1/K0 = 1.6 Vs/cm2 to 20 eV at 1/K0 = 0.6 Vs/cm2. For all experiments, TIMS elution voltages were calibrated linearly to obtain the reduced ion mobility coefficients (1/K0) using three Agilent ESI-L Tuning Mix ions (m/z, 1/K0: 622.0289, 0.9848 Vs/cm2; 922.0097, 1.1895 Vs/cm2; and 1221.9906, 1.3820 Vs/cm2).

Data processing

Spectra deconvolution, protein identification and relative quantification was performed using DIA-NN 1.8 (Data-Independent Acquisition by Neural Networks) [Demichev V. et al., Nat Methods. 2020; 17(1): 41–44, doi: 10.1038/s41592-019-0638-x]. Peptide search was performed in library-free mode, meaning that deep learning was used to generate a new *in silico* spectral library from the Uniprot reference proteome available for each species (downloaded on Sept 13th, 2021). The following settings were applied: output was filtered at 0.01 FDR, minimum fragment m/z was set to 200, maximum fragment m/z was set to 1800, N-terminal methionine excision was enabled, *in silico* digest involved cuts at K*,R*, maximum number of missed cleavages was set to 1, minimum peptide length was set to 7, maximum peptide length was set to 30, minimum precursor m/z was set to 350, maximum precursor m/z was set to 1300, minimum precursor charge was set to 1, maximum precursor charge was set to 4, cysteine carbamidomethylation was enabled as a fixed modification, scan window radius was set to 10, thread number was set to 40, mass accuracy

was fixed to 1e-05 (MS2) and 1e-05 (MS1). Finally, data were processed in R by filtering proteotypic precursors at sample fraction 80% within all samples of the same species, and a minimum of 3 precursors were used for protein quantification. The minimum threshold for Global.Q.Value, Global.PG.Q.Value, Q.Value, PG.Q.Value was set to 0.01. Precursors were summarized using the MaxLFQ algorithm. On average, we quantified 1,921 proteins per species, and obtained a low CV for technical replicates (median CV = 12.4%), indicating the low technical variation in the proteomes. The mass spectrometry proteomics data have been deposited to the ProteomeXchange Consortium via the PRIDE[78] partner repository with the dataset identifier PXD064343.

Mapping structures within an orthogroup

To generate a one-on-one assignment of the amino acids between different structures within an orthogroup, we utilised the structures that were aligned to the reference structure of *S. cer.*. In the first step we spanned a tree based on the euclidean distances between the C_α-atoms of the reference structure and the C_α-atoms of the aligned structures. Using a cut-off of 2 Å, we generated a C_α mapping matrix for each orthogroup. Each amino acid that could not be mapped to the reference structure was excluded from later analysis. An example of the mapping is shown Figure ED1f. Based on this mapping matrix, structural information as described below were projected onto the alignments. To calculate the mapping rate, the portion of residues that could be mapped to the reference structure versus the length of the reference structure was calculated. The mapped length was calculated as the number of amino acids of the original protein chain that could be mapped to the reference structure. The conservation ratio denotes the portion of no amino acid changes with respect to the corresponding reference structure divided by the length of the mapped parts. Over all analysed orthogroups a median CR of 62.9% (IQR: [53.6%, 71.2%], total: [24.5% to 89.2%]) was observed and the range was 24.5% to 89.2% (10% quantile: 47.8%, 90% quantile: 77.2%).

Binding site extraction

Binding sites were defined in two different ways. For the direct coordination sphere the binding site (and for the enrichment analysis also the active sites) were downloaded from UniProt in November 2022 [79]. To account for the physicochemical environment, we used crystal structures extracted from RCSB Protein Data Bank (RSCB.org) [80] and the crystal structure annotation of Yeastmine (http://yeastmine.yeastgenome.org/ accessed 7 Oct, 2021) [60]. Each structure was

aligned to the corresponding reference structure and all hetero-atoms were extracted. To remove crystalizing agents we removed halides and small anions like nitrate, azide, sulphate and phosphate as well as alkali metals, small cations like ammonium and heavy metals. In addition, water molecules were removed as well. All other heteroatoms were treated as ligands. To determine the binding site and its physicochemical environment, we selected all amino acids where at least one atom was within a distance of 5 Å to bound ligands or cofactors from X-ray crystallographic and cryo-EM structures from *S. cerevisiae* in our structural alignments.

Protein-protein interaction site extraction

Coordination sites were extracted for 106 of our reference structures based on 546 experimentally determined structures in PDB. The predicted structure of *Saccharomyces cerevisiae* was aligned to the respective crystal structures. For the extraction of the protein-protein interaction sites the same approach as for the binding site identification was used treating the second protein chain formally as a ligand. To obtain an overall representation for each orthogroup, we collected the obtained protein-protein interaction sites within an orthogroup into one binary vector. To calculate the mapping ratio, we used the mean mapping ratio over all reference crystal structures within an orthogroup.

Structural information extraction

Estimating properties and enzyme cost

All properties were estimated on the re-clustered orthogroups. Thus, orthogroups containing more than one cluster were split. In addition, the analysis was performed for every single reference structure in *S. cer.*.

The solvent accessible surface area (SASA) was evaluated using the Shrake-Rupley algorithm [81], the summarised secondary structure estimates were determined using the DSSP algorithm [82] as implemented in *MDTraj* 1.9.7 [83]. The pLDDT values were extracted from the predicted AlphaFold2 structures. For the relative amino acid content the ratio between a specific amino acid in the structure and the overall length of the protein chain was calculated. Thus, the relative amino acid content per structure over all amino acids sums up to one. To take into account other physicochemical properties we also included the amino acid hydrophobicity

(https://www.genome.jp/entry/aaindex:CIDH920105) [84] and other amino acid specific properties like the isoelectric point or the amino acid polarity [85] of the whole protein chain.

For estimating the protein costs, we included several different cost terms: Molecular weight [36], number of reaction steps [37], energy equivalents [37–40], glucose usage in response to perturbation of amino acid requirements in the context of metabolic flux models (FBA) [39,41], and costs of generating the enzymes required to synthesise the protein assuming maximal k_{cat} values (Protein Cost, k_{max}) [41]. For calculating an averaged normalised cost, each cost metric was bound between 0 and 1 and the mean per amino acid was calculated.

Properties were estimated for the whole non-aligned structures, the reference structure only and certain extracted structural features based on the amino acids that could be mapped to the reference structure (see, following subsection). For the calculation of the average properties per orthogroup only the structures belonging to the cluster of the reference structure as described above were used.

Extracted Structural features

To utilise one advantage of our structural predictions, we calculated most of the estimated properties not only for the mapped parts, but also for specific structural features. For this the mapped structures were divided into groups using different subset. For the secondary structural elements we used the summarised DSSP information to distinguish helical (H), extended (E) and random coil (C) structural elements. To determine tight turns, we checked for DSSP information of the form [HE][HE]Cx[HE][HE] varying x between 2 and 6 [45]. To distinguish the surface and core residues we calculated the maximal SASA per amino acid for non-terminal amino acids. For this we constructed an artificial helical protein chain GX1GX2G[...]GX20G with torsion angles \phi = -50° and \psi = 45° to achieve the maximal surface accessibility per amino acid [86]. Core residues were defined as buried residues with a maximal relative accessible surface area of 25% [87,88]. For the binding sites the definitions as given above were used. Also the crystal structure exclusive part of the binding site that was not defined in UniProt was extracted. In addition, the active side definition extracted from UniProt was included. Furthermore, positions of reference structure matching, fully conservation as well as positions with at least one amino acid or amino acid type mutation were tested. Another subset for amino acids with an pLDDT greater than 0.7 was estimated. As a last selection the intracellular location [89] was included splitting into membrane-

bound, cytosolic, luminal/extra-cellular and signalling as well as into a combination of cytosolic and luminal/extra-cellular.

Grouping of amino acids

For the grouping of amino acids we decided into 7 different groups. The 5 main groups were divided by physicochemical properties: (I) Acidic amino acids (D, E), basic amino acids (H, K, R), polar amino acids (C, N, Q, S, T), apolar amino acids (A, I, L, M, V) and aromatic amino acids (F, W, Y). The canonical amino acids G and P were treated as separate groups due to their unique backbone dynamics. In the case of G (flexible) a higher backbone flexibility can be achieved due to the missing side chain. In the case of P (break) a decreased backbone flexibility as well as influence on neighbouring amino acids are observed due to the ring formation of the side chain with the amino acid backbone. For calculating an amino acid type CR, changes between the 7 groups were considered.

Evaluated experimental data sets and databases

To investigate the relationship of structural evolution and experimental observables, we investigated several experimental data sets. In addition we included different databases for system level analysis. A summary of the sources used is found in Supplementary Table 7.1.

Flux and kcat calculations

For flux calculation genome-scale metabolic models for 329 yeast species were utilised [16]. A total of 66 conditions, encompassing various carbon sources combined with minimal media, were employed as inputs for the simulations. The maximum uptake rate for carbon sources was defined by setting the lower bound at -1 Cmmol/gDW/h. Growth was selected as the objective function for optimization. For each orthogroup, the mean fluxes across all conditions, excluding zero, were calculated to determine the average flux for each OG in each species. For the calculation of the number of species with Flux > 0, all 329 species were included. To compare calculated flux with previously measured ^{13}C flux, we used data from 7 different datasets extracted from 6 studies [90–95].

For the estimation of *kcat* values, we utilised species-specific models for various yeast species. Each model was meticulously refined, with reversible enzymatic reactions split into forward and

backward processes and reactions catalysed by isoenzymes segmented into separate reactions with individual enzyme complexes. SMILES information for substrates were extracted by mapping substrates into MetaNetX database [96]. Enzyme information, extracted from the model's gene-reaction rules (grRules), was complemented by protein sequences obtained via protein IDs from species-specific protein FASTA files. This dataset, encompassing reaction IDs, substrate names, SMILES information, and protein IDs, formed the basis for our deep learning kcat prediction model, DLKcat. Entries with non-specific SMILES or asterisks (*) in protein sequences were excluded from prediction. Since each enzyme can utilise multiple substrates in multiple reactions we first evaluated summarization within the reactions followed by the reactions per enzyme itself by different methods (maximal value, minimal value, mean or median). On orthogroup level in addition the standard variation as well as the coefficient of variation were calculated. In the course of the manuscript we only report the (within and between reactions per enzyme) median summarised log2-transformed values.

Enrichment analysis

For categorical data collections such as gene locus, enzyme class, metabolic pathway or GO slim terms we performed an enrichment if not mentioned otherwise of the top 25 % as well as lowest 25 % of the analysed observables. To get a more detailed understanding we analysed enzyme classes on the first level as well as combinations of the first and the second or third level respectively. For the enrichment analysis we performed a Fisher's exact test as implemented in *scipy 1.8.1* [97]. Within each collection, we performed Benjamini-Hochberg multiple testing correction [98] as implemented in *statsmodels 0.13.2* [99]. The significance level was set to adj. p-value < 0.05. To account for the analysed protein space, we have only included the analysed orthogroups as a background truth. In addition, we calculated the area under the receiver operating characteristic curve (abbrev. AUC) as implemented in *scikit-learn 1.1.1* [100]. For the calculation of the enrichment factor, we divided the number of observations by the number of expected observations assuming a random distribution.

Correlation and statistical analysis

For statistical testing as well as correlation calculations *scipy 1.8.1* [97] was used. Depending on the analysis we distinguished between the linear Pearson correlation coefficient r, the rank-order Spearman correlation coefficient ρ [101] or for discrete data Kendall's τ coefficient [102,103] using

variant="b". To account for multiple testing, the data set was split into data extracted from the structural alignment and experimental data. Benjamini-Hochberg multiple testing correction [98] was applied as implemented in *statsmodels 0.13.2* [99] for all calculated p-values within the same data, the same correlation measure and the same property (noted on the x-axis of the respective plots). For statistical testing we used the nonparametric two-sided Wilcoxon-Mann-Whitney test [104,105] for non-paired samples as well as the nonparametric two-sided Wilcoxon signed-rank test [105] for paired samples. For comparing two continuous distributions we used the two-sided, two-sample Kolmogorov-Smirnov test [106]. In all cases missing values were omitted. For the visualisation of distributions a smoothing function with a smoothing-factor ≤ 1E-8 was applied, which might cause artefacts at the borders of the distributions. For comparing the performance of the machine learning algorithm on a statistical level, we calculated the average predicted probability per amino acid. We assigned each fully conserved amino acid to either the group of binding-site or the group of non-binding site residues. We compared both groups using a two-sided Wilcoxon signed-rank test.

For calculating the effect sizes we used Cliff's Δ [107,108], which accounts for the distribution of signs between all data points (unpaired data). The value is bound in [-1,1].

$$Cliff's\ \Delta = \frac{\sum_{i=1}^{N} \sum_{j=1}^{M} \delta_{ij}}{N \cdot M} \quad (4)$$

with δ_{ij} denoting -1 for a negative difference and +1 for positive differences between two data point $x_{i,a}$ and $x_{j,b}$ from two samples *a* and *b* with sample sizes *N* and *M*. For paired samples we analyzed only the sign difference of the pairs.

$$Cliff's\ \Delta = \frac{\sum_{i=1}^{N} \delta_{ii}}{N} \quad (5)$$

with δ_{ii} comparing two data point $x_{i,a}$ and $x_{i,b}$ from two samples *a* and *b* with sample sizes *N.*

Network clustering

To extract regions that never changed during evolution, within each orthogroup, we first identified all fully conserved residues in the alignment. In a next step we set up a network of these residues using *igraph 0.9.9* [109] in which nodes are connected if the residue's C_α-atoms are not more then 10 Å apart. Using a fixed seed of 42, we clustered the network using the Leiden algorithm [110] as

implemented in *leidenalg 0.8.9* using *CPMVertexPartition* and a resolution parameter of 0.05. The clusters were extracted and plotted using *igraph*. To remove artefacts, the minimum number of amino acids per cluster was set to 5. For network representations the sizes were normalized and shifted by a constant value for better representation of nodes with lower original size.

Machine learning

To increase the sample size we used different parameter combinations for the network clustering: Distance for tree-based neighbor-search for network creation of 8, 9, 10, 11 or 12 Å; clustering resolution of 0.05 or 0.1; modularity turned on or off as well as different seeds (42, 1337 and 31415). Duplicated clusters were removed and cluster size was limited between [5,25]. As features, physico chemical properties (as described above), the amino acid constitution and classification as well as the SASA and the surface-to-core ratio were used. To label the clusters the UniProt binding site definitions were used. A cluster was assigned to contain a binding site if at least 10 % of the residues belonged to an annotated binding site. Only reference structures with known binding sites were considered. This results in 24,434 labelled samples with a label ratio of 0.29.

For setting up machine learning we used the *HistGradientBoostingClassifier()* as implemented in *sci-kit learn 1.5.1* [100] with balanced class-weights. For reproducibility, the overall seed was fixed to 42. The data were split using a 5-fold stratified-group-split. As a group the orthogroup was used to prevent any memory leakage. Each split was subsequently used as a testing set, whereas the other four splits were used to train the model. Within each training cycle the parameter space was optimized by varying the *max_depth* in [2,3,4], the *l2_regularization* in [1,10,20,50,100] and *max_iter* in [100,250,500] using a randomized 4-fold stratified-grouped cross validation. To prevent overfitting a refitting procedure based on the balanced accuracy was used, that optimizes the balance accuracy and neglects performance differences between training and validation larger than 0.2. Splits that do not pass the refitting were rejected. Performance was evaluated based on the left-out test set. To obtain a broader estimate, the machine learning setup was repeated 10-times. For reproducibility the seed for each run was fixed to a random seed between 1 and 10,000 given the overall seed for the random number generation. For an overall estimate the micro- and macro-average over all test-set performances were calculated.

To obtain a random control, we decided to use two different approaches. The first approach randomized the binding site annotation assuming the same overlap between binding site and fully

conserved residues as the reference. The second approach randomized the labels, preserving the class-distribution.

Structural representation

For the evaluation of the positions of the positive selected residues, we centred the CIII-CIV-complex and rotated it onto the principal axis using *GROMACS 2021.5* [111]. We extracted the y-coordinates as they resemble the orientation of the membrane-inserted complex. For structural visualisations we used *VMD 1.9.4*. *VMD* is developed with NIH support by the Theoretical and Computational Biophysics group at the Beckman Institute, University of Illinois at Urbana-Champaign. During the course of the manuscript we used the following experimentally determined structures, 1A3W(*S. cerevisiae* Cdc19p)[112], 5Y9D(*Y. lipolytica* ACO1) [113], 3PYM (*S. cereviiae TDH3*), 5YJA (*Streptococcus agalactiae* GAPDH)[114], 6HU9 (Cytochrome-c-oxidase/cytochrome bc1 complex) [59].

Evolution rate (dN/dS) calculations

For evolution rate (dN/dS) calculations, nucleotide alignments were required. Nucleotide sequences were obtained from either model organism databases (*C. albicans* and *S. cerevisiae*), NCBI's datasets resource [https://www.ncbi.nlm.nih.gov/datasets/], NCBI's genome database by downloading data from individual contigs, or [2]. These sequences were then threaded onto protein alignments using *phykit v1.11.15* [115]. The alignments were then strictly trimmed using *clipkit v1.4.1* with options *-m gappy -g clipkit* [72]. Three refined orthogroups for which the strict trimming was less than 25% of the median sequence length were removed at this stage, OG1306_REF_Scer_AF-P38298-F1-model_v2, OG1746_REF_Scer_AF-P32642-F1-model_v2, and OG2228_geotrichum_candidum__OG2228__43_6293. Strictly trimmed alignments, and phylogenetic trees when necessary, were then formatted and sequences given shorter names in order to work with CodeML.

DN/DS was calculated using the *codeml* function from *PAML v4.10.6* [116]. Two strategies were used to estimate average dN/dS values for entire orthogroups. The Yang and Nielsen method (YN00) method [117] and the one-ratio model (M0) [52,53], which is informed by phylogenetic trees. Values from the one-ratio model were used for enrichment calculations, and a filter with dS>3 was applied as described in Supplementary Note 6.

To estimate dN/dS values at the residue level, site models which assume either neutral drift (M1a) or positive selection (M2a) were compared [54–56]. Data was calculated for 398 orthogroups, 295 that had dS<3, and of those, 270 had no convergence issue and were calculated for all tests. Of those 270 orthogroups, only three performed better ($\chi 2>5.99$) under a model of positive selection than under a model of neutral drift, those containing Tdh1/2/3p (OG1145), Fum1p (OG1218), and TPI1p (OG2197). Site classes were assigned based on the Naive Empirical Bayes method [54] for each residue in trimmed alignments.

To identify dN/dS estimates at the branch level for example orthogroups containing Pox1p, GAPDH (Tdh1/2/3p) and proteins from the ETC, we followed a two-stage process. First, we used the free ratio branch model (M1) [53,57]. Any branches for which dN/dS was greater than 1.1 in that test, were then tested using the branch-site test for positive selection (BS) [56,58]. If the $\chi 2$ threshold between model A and model A with ω fixed at 1 was greater than 3.84, then residues for which the Bayes Empirical Bayes posterior probability of dN/dS>1 was greater than 0.95 were considered under positive selection.

1. Opulente, D. A. *et al.* Genomic factors shape carbon and nitrogen metabolic niche breadth across Saccharomycotina yeasts. *Science* **384**, eadj4503 (2024).
2. Shen, X.-X. *et al.* Tempo and Mode of Genome Evolution in the Budding Yeast Subphylum. *Cell* **175**, 1533-1545.e20 (2018).
3. Hagman, A., Säll, T. & Piškur, J. Analysis of the yeast short-term Crabtree effect and its origin. *FEBS J.* **281**, 4805–4814 (2014).
4. Hagman, A. & Piškur, J. A study on the fundamental mechanism and the evolutionary driving forces behind aerobic fermentation in yeast. *PloS One* **10**, e0116942 (2015).
5. Marcet-Houben, M. & Gabaldón, T. Beyond the Whole-Genome Duplication: Phylogenetic Evidence for an Ancient Interspecies Hybridization in the Baker's Yeast Lineage. *PLOS Biol.* **13**, e1002220 (2015).
6. Gonçalves, C. *et al.* Evidence for loss and reacquisition of alcoholic fermentation in a fructophilic yeast lineage. *eLife* **7**, e33034 (2018).
7. Gonçalves, P. & Gonçalves, C. Horizontal gene transfer in yeasts. *Curr. Opin. Genet. Dev.* **76**, 101950 (2022).
8. Kominek, J. *et al.* Eukaryotic Acquisition of a Bacterial Operon. *Cell* **176**, 1356-1366.e10 (2019).
9. Marsit, S. *et al.* Evolutionary Advantage Conferred by an Eukaryote-to-Eukaryote Gene Transfer Event in Wine Yeasts. *Mol. Biol. Evol.* **32**, 1695–1707 (2015).
10. Kurtzman, C., Fell, J. W. & Boekhout, T. *The Yeasts: A Taxonomic Study*. (Elsevier, 2011).
11. Riley, R. *et al.* Comparative genomics of biotechnologically important yeasts. *Proc. Natl. Acad. Sci. U. S. A.* **113**, 9882–9887 (2016).
12. Steenwyk, J. L. *et al.* An orthologous gene coevolution network provides insight into eukaryotic cellular and genomic structure and function. *Sci. Adv.* **8**, eabn0105 (2022).
13. Wolters, J. F., LaBella, A. L., Opulente, D. A., Rokas, A. & Hittinger, C. T. Mitochondrial genome diversity across the subphylum Saccharomycotina. *Front. Microbiol.* **14**, 1268944

(2023).

14. Wu, J. *et al.* Yeast diversity in relation to the production of fuels and chemicals. *Sci. Rep.* **7**, (2017).

15. Li, F. *et al.* Deep learning-based kcat prediction enables improved enzyme-constrained model reconstruction. *Nat. Catal.* 1–11 (2022) doi:10.1038/s41929-022-00798-z.

16. Lu, H. *et al.* Yeast metabolic innovations emerged via expanded metabolic network and gene positive selection. *Mol. Syst. Biol.* **17**, e10427 (2021).

17. Terwilliger, T. C. *et al.* AlphaFold predictions are valuable hypotheses and accelerate but do not replace experimental structure determination. *Nat. Methods* **21**, 110–116 (2024).

18. Stevens, A. O. & He, Y. Benchmarking the Accuracy of AlphaFold 2 in Loop Structure Prediction. *Biomolecules* **12**, 985 (2022).

19. Jumper, J. *et al.* Highly accurate protein structure prediction with AlphaFold. *Nature* **596**, 583–589 (2021).

20. Abramson, J. *et al.* Accurate structure prediction of biomolecular interactions with AlphaFold 3. *Nature* 1–3 (2024) doi:10.1038/s41586-024-07487-w.

21. Tunyasuvunakool, K. *et al.* Highly accurate protein structure prediction for the human proteome. *Nature* **596**, 590–596 (2021).

22. Chakravarty, D. & Porter, L. L. ALPHAFOLD2 fails to predict protein fold switching. *Protein Sci.* **31**, e4353 (2022).

23. David, A., Islam, S., Tankhilevich, E. & Sternberg, M. J. E. The AlphaFold Database of Protein Structures: A Biologist’s Guide. *J. Mol. Biol.* **434**, 167336 (2022).

24. Ruff, K. M. & Pappu, R. V. AlphaFold and Implications for Intrinsically Disordered Proteins. *J. Mol. Biol.* **433**, 167208 (2021).

25. Bondos, S. E., Dunker, A. K. & Uversky, V. N. On the roles of intrinsically disordered proteins and regions in cell communication and signaling. *Cell Commun. Signal. CCS* **19**, 88 (2021).

26. DeForte, S. & Uversky, V. N. Not an exception to the rule: the functional significance of intrinsically disordered protein regions in enzymes. *Mol. Biosyst.* **13**, 463–469 (2017).

27. Evans, R. *et al.* Protein complex prediction with AlphaFold-Multimer. 2021.10.04.463034 Preprint at https://doi.org/10.1101/2021.10.04.463034 (2022).

28. Kwak, S. & Jin, Y.-S. Production of fuels and chemicals from xylose by engineered Saccharomyces cerevisiae: a review and perspective. *Microb. Cell Factories* **16**, 82 (2017).

29. Wei, S. *et al.* A Thi2p Regulatory Network Controls the Post-glucose Effect of Xylose Utilization in Saccharomyces cerevisiae. *Front. Microbiol.* **10**, (2019).

30. Whitworth, D. A. & Ratledge, C. Phosphoketolase in Rhodotorula graminis and Other Yeasts. *Microbiology* **102**, 397–401 (1977).

31. Ingram-Smith, C., Martin, S. R. & Smith, K. S. Acetate kinase: not just a bacterial enzyme. *Trends Microbiol.* **14**, 249–253 (2006).

32. Myers, K. S. *et al.* Rewired cellular signaling coordinates sugar and hypoxic responses for anaerobic xylose fermentation in yeast. *PLOS Genet.* **15**, e1008037 (2019).

33. Yu, J. S. L. *et al.* Inorganic sulfur fixation via a new homocysteine synthase allows yeast cells to cooperatively compensate for methionine auxotrophy. *PLoS Biol.* **20**, e3001912 (2022).

34. Sonal, Yuan, A. E., Yang, X. & Shou, W. When is an auxotroph not an auxotroph: how budding yeast lacking MET17 collectively overcome their metabolic defect. 2023.05.18.541364 Preprint at https://doi.org/10.1101/2023.05.18.541364 (2023).

35. Oss, S. B. V. *et al.* Unexpected growth of a classic yeast auxotroph. 2022.01.19.476918 Preprint at https://doi.org/10.1101/2022.01.19.476918 (2022).

36. Seligmann, H. Cost-minimization of amino acid usage. *J. Mol. Evol.* **56**, 151–161 (2003).

37. Craig, C. L. & Weber, R. S. Selection costs of amino acid substitutions in ColE1 and ColIa gene clusters harbored by Escherichia coli. *Mol. Biol. Evol.* **15**, 774–776 (1998).

38. Akashi, H. & Gojobori, T. Metabolic efficiency and amino acid composition in the

proteomes of Escherichia coli and Bacillus subtilis. *Proc. Natl. Acad. Sci. U. S. A.* **99**, 3695–3700 (2002).

39. Barton, M. D., Delneri, D., Oliver, S. G., Rattray, M. & Bergman, C. M. Evolutionary Systems Biology of Amino Acid Biosynthetic Cost in Yeast. *PLOS ONE* **5**, e11935 (2010).
40. Wagner, A. Energy constraints on the evolution of gene expression. *Mol. Biol. Evol.* **22**, 1365–1374 (2005).
41. Chen, Y. & Nielsen, J. Yeast has evolved to minimize protein resource cost for synthesizing amino acids. *Proc. Natl. Acad. Sci.* **119**, e2114622119 (2022).
42. Choi, Y. S., Yang, J.-S., Choi, Y., Ryu, S. H. & Kim, S. Evolutionary conservation in multiple faces of protein interaction. *Proteins Struct. Funct. Bioinforma.* **77**, 14–25 (2009).
43. Chakrabartty, A., Kortemme, T. & Baldwin, R. L. Helix propensities of the amino acids measured in alanine-based peptides without helix-stabilizing side-chain interactions. *Protein Sci.* **3**, 843–852 (1994).
44. Ho, B. K. & Brasseur, R. The Ramachandran plots of glycine and pre-proline. *BMC Struct. Biol.* **5**, 14 (2005).
45. Chou, K.-C. Prediction of Tight Turns and Their Types in Proteins. *Anal. Biochem.* **286**, 1–16 (2000).
46. Kyte, J. & Doolittle, R. F. A simple method for displaying the hydropathic character of a protein. *J. Mol. Biol.* **157**, 105–132 (1982).
47. White, M. E. H., Gil, J. & Tate, E. W. Proteome-wide structural analysis identifies warhead- and coverage-specific biases in cysteine-focused chemoproteomics. *Cell Chem. Biol.* **30**, 828-838.e4 (2023).
48. Marino, S. M. & Gladyshev, V. N. Cysteine Function Governs Its Conservation and Degeneration and Restricts Its Utilization on Protein Surfaces. *J. Mol. Biol.* **404**, 902–916 (2010).
49. Vitalini, F., Noé, F. & Keller, B. G. A Basis Set for Peptides for the Variational Approach to

Conformational Kinetics. *J. Chem. Theory Comput.* **11**, 3992–4004 (2015).

50. Kimura, M. Preponderance of synonymous changes as evidence for the neutral theory of molecular evolution. *Nature* **267**, 275–276 (1977).
51. Miyata, T. & Yasunaga, T. Molecular evolution of mRNA: a method for estimating evolutionary rates of synonymous and amino acid substitutions from homologous nucleotide sequences and its application. *J. Mol. Evol.* **16**, 23–36 (1980).
52. Goldman, N. & Yang, Z. A codon-based model of nucleotide substitution for protein-coding DNA sequences. *Mol. Biol. Evol.* **11**, 725–736 (1994).
53. Yang, Z. & Nielsen, R. Synonymous and nonsynonymous rate variation in nuclear genes of mammals. *J. Mol. Evol.* **46**, 409–418 (1998).
54. Nielsen, R. & Yang, Z. Likelihood models for detecting positively selected amino acid sites and applications to the HIV-1 envelope gene. *Genetics* **148**, 929–936 (1998).
55. Wong, W. S. W., Yang, Z., Goldman, N. & Nielsen, R. Accuracy and Power of Statistical Methods for Detecting Adaptive Evolution in Protein Coding Sequences and for Identifying Positively Selected Sites. *Genetics* **168**, 1041–1051 (2004).
56. Yang, Z., Wong, W. S. W. & Nielsen, R. Bayes Empirical Bayes Inference of Amino Acid Sites Under Positive Selection. *Mol. Biol. Evol.* **22**, 1107–1118 (2005).
57. Yang, Z. Likelihood ratio tests for detecting positive selection and application to primate lysozyme evolution. *Mol. Biol. Evol.* **15**, 568–573 (1998).
58. Zhang, J., Nielsen, R. & Yang, Z. Evaluation of an Improved Branch-Site Likelihood Method for Detecting Positive Selection at the Molecular Level. *Mol. Biol. Evol.* **22**, 2472–2479 (2005).
59. Hartley, A. M. *et al.* Structure of yeast cytochrome c oxidase in a supercomplex with cytochrome bc1. *Nat. Struct. Mol. Biol.* **26**, 78–83 (2019).
60. Wong, E. D. *et al.* Saccharomyces genome database update: server architecture, pan-genome nomenclature, and external resources. *Genetics* **224**, iyac191 (2023).

61. YeastPathways Database Website Home. https://pathway.yeastgenome.org/ (2023).
62. Eisenberg, A. R. *et al.* Translation Initiation Site Profiling Reveals Widespread Synthesis of Non-AUG-Initiated Protein Isoforms in Yeast. *Cell Syst.* **11**, 145-160.e5 (2020).
63. Almagro Armenteros, J. J. *et al.* Detecting sequence signals in targeting peptides using deep learning. *Life Sci. Alliance* **2**, e201900429 (2019).
64. Sharma, S. & Schiller, M. R. The carboxy-terminus, a key regulator of protein function. *Crit. Rev. Biochem. Mol. Biol.* **54**, 85–102 (2019).
65. Goddard, T. D. *et al.* UCSF ChimeraX: Meeting modern challenges in visualization and analysis. *Protein Sci.* **27**, 14–25 (2018).
66. Meng, E. C. *et al.* UCSF ChimeraX: Tools for structure building and analysis. *Protein Sci.* **32**, e4792 (2023).
67. Meng, E. C., Pettersen, E. F., Couch, G. S., Huang, C. C. & Ferrin, T. E. Tools for integrated sequence-structure analysis with UCSF Chimera. *BMC Bioinformatics* **7**, 339 (2006).
68. Pettersen, E. F. *et al.* UCSF ChimeraX: Structure visualization for researchers, educators, and developers. *Protein Sci.* **30**, 70–82 (2021).
69. Needleman, S. B. & Wunsch, C. D. A general method applicable to the search for similarities in the amino acid sequence of two proteins. *J. Mol. Biol.* **48**, 443–453 (1970).
70. Zhang, C., Shine, M., Pyle, A. M. & Zhang, Y. US-align: universal structure alignments of proteins, nucleic acids, and macromolecular complexes. *Nat. Methods* **19**, 1109–1115 (2022).
71. Zhang, Y. & Skolnick, J. TM-align: a protein structure alignment algorithm based on the TM-score. *Nucleic Acids Res.* **33**, 2302–2309 (2005).
72. Steenwyk, J. L., Iii, T. J. B., Li, Y., Shen, X.-X. & Rokas, A. ClipKIT: A multiple sequence alignment trimming software for accurate phylogenomic inference. *PLOS Biol.* **18**, e3001007 (2020).

73. Minh, B. Q. *et al.* IQ-TREE 2: New Models and Efficient Methods for Phylogenetic Inference in the Genomic Era. *Mol. Biol. Evol.* **37**, 1530–1534 (2020).

74. Waterhouse, A. M., Procter, J. B., Martin, D. M. A., Clamp, M. & Barton, G. J. Jalview Version 2—a multiple sequence alignment editor and analysis workbench. *Bioinformatics* **25**, 1189–1191 (2009).

75. Darzi, Y., Letunic, I., Bork, P. & Yamada, T. iPath3.0: interactive pathways explorer v3. *Nucleic Acids Res.* **46**, W510–W513 (2018).

76. Olin-Sandoval, V. *et al.* Lysine harvesting is an antioxidant strategy and triggers underground polyamine metabolism. *Nature* **572**, 249–253 (2019).

77. Messner, C. B. *et al.* Ultra-fast proteomics with Scanning SWATH. *Nat. Biotechnol.* **39**, 846–854 (2021).

78. Perez-Riverol, Y. *et al.* The PRIDE database at 20 years: 2025 update. *Nucleic Acids Res.* **53**, D543–D553 (2025).

79. The UniProt Consortium. UniProt: the Universal Protein Knowledgebase in 2023. *Nucleic Acids Res.* **51**, D523–D531 (2023).

80. Berman, H. M. *et al.* The Protein Data Bank. *Nucleic Acids Res.* **28**, 235–242 (2000).

81. Shrake, A. & Rupley, J. A. Environment and exposure to solvent of protein atoms. Lysozyme and insulin. *J. Mol. Biol.* **79**, 351–371 (1973).

82. Kabsch, W. & Sander, C. Dictionary of protein secondary structure: pattern recognition of hydrogen-bonded and geometrical features. *Biopolymers* **22**, 2577–2637 (1983).

83. McGibbon, R. T. *et al.* MDTraj: A Modern Open Library for the Analysis of Molecular Dynamics Trajectories. *Biophys. J.* **109**, 1528–1532 (2015).

84. Cid, H., Bunster, M., Canales, M. & Gazitúa, F. Hydrophobicity and structural classes in proteins. *Protein Eng.* **5**, 373–375 (1992).

85. Gao, Z. *et al.* Hierarchical graph learning for protein–protein interaction. *Nat. Commun.* **14**, 1093 (2023).

86. Tien, M. Z., Meyer, A. G., Sydykova, D. K., Spielman, S. J. & Wilke, C. O. Maximum Allowed Solvent Accessibilites of Residues in Proteins. *PLOS ONE* **8**, e80635 (2013).

87. Gong, H. *et al.* Improving prediction of burial state of residues by exploiting correlation among residues. *BMC Bioinformatics* **18**, 70 (2017).

88. Momen-Roknabadi, A., Sadeghi, M., Pezeshk, H. & Marashi, S.-A. Impact of residue accessible surface area on the prediction of protein secondary structures. *BMC Bioinformatics* **9**, 357 (2008).

89. Weill, U., Cohen, N., Fadel, A., Ben-Dor, S. & Schuldiner, M. Protein topology prediction algorithms systematically investigated in the yeast Saccharomyces cerevisiae. *BioEssays News Rev. Mol. Cell. Dev. Biol.* **41**, e1800252 (2019).

90. Blank, L. M., Kuepfer, L. & Sauer, U. Large-scale 13C-flux analysis reveals mechanistic principles of metabolic network robustness to null mutations in yeast. *Genome Biol.* **6**, R49 (2005).

91. Daran-Lapujade, P. *et al.* Role of transcriptional regulation in controlling fluxes in central carbon metabolism of Saccharomyces cerevisiae. A chemostat culture study. *J. Biol. Chem.* **279**, 9125–9138 (2004).

92. Gombert, A. K., Moreira dos Santos, M., Christensen, B. & Nielsen, J. Network identification and flux quantification in the central metabolism of Saccharomyces cerevisiae under different conditions of glucose repression. *J. Bacteriol.* **183**, 1441–1451 (2001).

93. Jouhten, P. *et al.* Oxygen dependence of metabolic fluxes and energy generation of Saccharomyces cerevisiae CEN.PK113-1A. *BMC Syst. Biol.* **2**, 60 (2008).

94. Kajihata, S. *et al.* ^{13}C-based metabolic flux analysis of Saccharomyces cerevisiae with a reduced Crabtree effect. *J. Biosci. Bioeng.* **120**, 140–144 (2015).

95. Raghevendran, V., Gombert, A. K., Christensen, B., Kötter, P. & Nielsen, J. Phenotypic characterization of glucose repression mutants of Saccharomyces cerevisiae using experiments with 13C-labelled glucose. *Yeast Chichester Engl.* **21**, 769–779 (2004).

96. Moretti, S., Tran, V. D. T., Mehl, F., Ibberson, M. & Pagni, M. MetaNetX/MNXref: unified namespace for metabolites and biochemical reactions in the context of metabolic models. *Nucleic Acids Res.* **49**, D570–D574 (2021).

97. Virtanen, P. *et al.* SciPy 1.0: fundamental algorithms for scientific computing in Python. *Nat. Methods* **17**, 261–272 (2020).

98. Benjamini, Y. & Hochberg, Y. Controlling the False Discovery Rate: A Practical and Powerful Approach to Multiple Testing. *J. R. Stat. Soc. Ser. B Methodol.* **57**, 289–300 (1995).

99. Seabold, S. & Perktold, J. Statsmodels: Econometric and Statistical Modeling with Python. in 92–96 (Austin, Texas, 2010). doi:10.25080/Majora-92bf1922-011.

100. Pedregosa, F. *et al.* Scikit-learn: Machine Learning in Python. *J. Mach. Learn. Res.* **12**, 2825–2830 (2011).

101. Spearman, C. The Proof and Measurement of Association between Two Things. *Am. J. Psychol.* **15**, 72–101 (1904).

102. Kendall, M. G. The treatment of ties in ranking problems. *Biometrika* **33**, 239–251 (1945).

103. Kendall, M. G. A New Measure of Rank Correlation. *Biometrika* **30**, 81–93 (1938).

104. Mann, H. B. & Whitney, D. R. On a Test of Whether one of Two Random Variables is Stochastically Larger than the Other. *Ann. Math. Stat.* **18**, 50–60 (1947).

105. Wilcoxon, F. Individual Comparisons by Ranking Methods. *Biom. Bull.* **1**, 80–83 (1945).

106. Smirnov, N. Table for Estimating the Goodness of Fit of Empirical Distributions. *Ann. Math. Stat.* **19**, 279–281 (1948).

107. Cliff, N. Dominance statistics: Ordinal analyses to answer ordinal questions. *Psychol. Bull.* **114**, 494–509 (1993).

108. Macbeth, G., Razumiejczyk, E. & Ledesma, R. D. Cliff´s Delta Calculator: A non-parametric effect size program for two groups of observations. *Univ. Psychol.* **10**, 545–555 (2011).

109. Csárdi, G. & Nepusz, T. The igraph software package for complex network research. in (2006).

110. Traag, V. A., Waltman, L. & van Eck, N. J. From Louvain to Leiden: guaranteeing well-connected communities. *Sci. Rep.* **9**, 5233 (2019).

111. Abraham, M. J. *et al.* GROMACS: High performance molecular simulations through multi-level parallelism from laptops to supercomputers. *SoftwareX* **1–2**, 19–25 (2015).

112. Jurica, M. S. *et al.* The allosteric regulation of pyruvate kinase by fructose-1,6-bisphosphate. *Structure* **6**, 195–210 (1998).

113. Kim, S. & Kim, K.-J. Structural insight into the substrate specificity of acyl-CoA oxidase1 from Yarrowia lipolytica for short-chain dicarboxylyl-CoAs. *Biochem. Biophys. Res. Commun.* **495**, 1628–1634 (2018).

114. Schormann, N. *et al.* Crystal Structures of Group B Streptococcus Glyceraldehyde-3-Phosphate Dehydrogenase: Apo-Form, Binary and Ternary Complexes. *PLOS ONE* **11**, e0165917 (2016).

115. Steenwyk, J. L. *et al.* PhyKIT: a broadly applicable UNIX shell toolkit for processing and analyzing phylogenomic data. *Bioinformatics* **37**, 2325–2331 (2021).

116. Yang, Z. PAML 4: phylogenetic analysis by maximum likelihood. *Mol. Biol. Evol.* **24**, 1586–1591 (2007).

117. Yang, Z. & Nielsen, R. Estimating Synonymous and Nonsynonymous Substitution Rates Under Realistic Evolutionary Models. *Mol. Biol. Evol.* **17**, 32–43 (2000).
