# Supplementary figures and images for: The role of metabolism in shaping enzyme structures over 400 million years
Source: Nature. 2025 Jul 9;644(8075):280–9. doi: 10.1038/s41586-025-09205-6 (PMC12328220; doi:10.1038/s41586-025-09205-6)

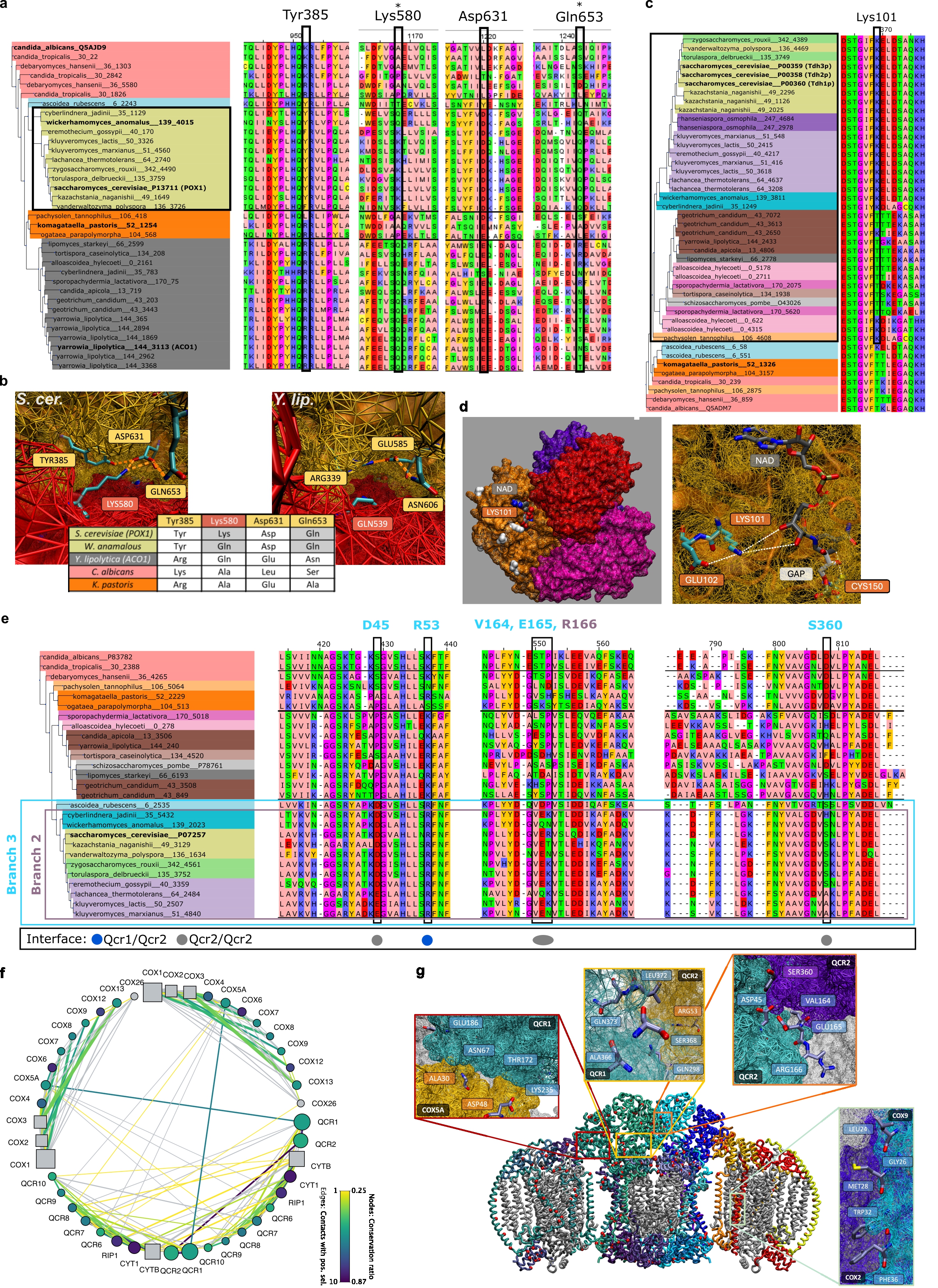

Supplement: Supplementary file 18 — Source Data Extended Data Fig. 9 [file 41586_2025_9205_MOESM18_ESM.jpg]
